# Student Burnout in Children and Adolescents: The Role of Attachment and Emotion Regulation

**DOI:** 10.3390/children10091443

**Published:** 2023-08-24

**Authors:** Ioana Alexandra Iuga, Oana Alexandra David, Marie Danet

**Affiliations:** 1 Evidence-Based Psychological Assessment and Interventions Doctoral School, Babeş-Bolyai University, No. 37 Republicii Street, 400015 Cluj-Napoca, Romania; ioana.iuga@ubbcluj.ro; 2 DATA Lab, The International Institute for the Advanced Studies of Psychotherapy and Applied Mental Health, Babes-Bolyai University, No. 37 Republicii Street, 400015 Cluj-Napoca, Romania; 3 Department of Clinical Psychology and Psychotherapy, Babeş-Bolyai University, No. 37 Republicii Street, 400015 Cluj-Napoca, Romania; 4 Department of Psychology, University of Lille, No. 42 Paul Duez Street, 59000 Lille, France; marie.danet@univ-lille.fr; 5 University of Lille, ULR 4072—PSITEC—Psychologie: Interactions, Temps, Emotions, Cognitions, 59000 Lille, France

**Keywords:** attachment security, gender differences, middle school, regulation strategies, school burnout

## Abstract

In an effort to explain the factors contributing to the development of student burnout, a construct that has received attention in relation to academic outcomes, including burnout, is emotion regulation. Further, attachment theory has been used to explore the variations in the use of particular emotion regulation strategies, and attachment has received support as a contributing factor. The aim of the study is to explore the role of attachment security and emotion regulation strategies associated with student burnout symptoms in a sample of 602 Romanian children and adolescents (55% female) aged 8–16 (M = 10.45) from 18 schools. A secondary objective was to explore the gender differences in burnout symptoms. The results show that attachment security negatively predicts student burnout symptoms. Further, a higher attachment security positively predicts the use of adaptive emotion regulation strategies, which, in turn, are negatively related to student burnout. Emotion regulation strategies mediate the relationship between attachment and burnout symptoms. No gender differences have been identified. The study has practical implications for both parents and specialists, bringing to attention the importance of secure attachment in children, which could further encourage the use of adaptive emotion regulation strategies.

## 1. Introduction

The term burnout has been used to label the “psychological syndromes of emotional exhaustion, depersonalization, and reduced personal accomplishment that can occur among individuals who work with other people in some capacity” [1]. Burnout was originally explored in the human services and care professions [2], as well as in highly stressful professions such as pilots and police officers [3,4]. Recently, promising data show the possibility of burnout symptoms manifesting themselves in a wider range of contexts in the lives of people of all ages and occupations, including housewives, informal caregivers, and students [5,6,7]. 

Student burnout is important for several reasons. On an individual student level, burnout has been associated with a range of mental health issues and challenges in adaptation. These include heightened depressive symptoms, diminished life satisfaction, disrupted sleep quality, physical complaints, and academic paths characterized by a greater likelihood of school abandonment and reduced academic performance [8,9,10]. From a policy perspective, investigating this subject aids experts in comprehending student behaviors by examining how burnout impacts students’ engagement with the institution and their interactions with peers and faculty.

According to Salmela-Aro et al. [11], school burnout can be understood as an outcome arising from inadequately handled stress associated with educational responsibilities. This is reflected in the form of exhaustion due to academic demands, developing a cynical attitude towards studying, and experiencing diminished effectiveness when engaging with school-related tasks. Secondary education students encounter notable stress levels, as evidenced by a comprehensive survey undertaken by the Organization for Economic Co-operation and Development (OECD) across 72 nations. The survey’s findings indicate that a substantial 66% of students disclosed experiencing stress attributed to unsatisfactory grades, while an additional 59% reported feeling stressed due to evaluation circumstances [12]. Estimating the prevalence of school burnout is made difficult by the lack of consensus related to its diagnostic criteria [13] and a variability in burnout definitions, assessment methods, and study quality. A study conducted on a large sample of Slovenian youth aged 15 to 19 shows that 6.7% of the students experience a high level of burnout [14], while a study conducted in a French sample showed that over 40% of students presented burnout symptoms that have the same manifestations to the ones common in professional contexts [15]. 

Originally confined to the workplace, the concept of burnout has extended its scope to encompass not only university students but also secondary and high school students. The change resulted in the application of several theories that had previously been used to explain burnout at work and are now being used to explain burnout in the classroom. These theories include the Existential Perspective [16], Social Cognitive Theory [17], Demands-Resources Model [18], and Conservation of Resources Theory [19]. The knowledge of burnout has grown along this development to include many educational situations, providing insights into its underlying mechanisms and effects on students.

According to the Conservation of Resources (COR) theory, there are four distinct types of resources that can predict optimal functioning [19]. In the educational context, these resources may encompass a variety of things, including material possessions (i.e., books, technology, and personal belongings), favorable circumstances (i.e., having a stable and supportive environment, access to educational opportunities, positive relationships with peers and educators, and a conducive learning atmosphere), character traits (i.e., self-confidence, self-esteem, resilience, and adaptability), and energy sources (i.e., financial resources, access to loans, social support networks, and other forms of assistance). A surplus of resources contributes to a reduced perception of demands as overwhelming, facilitating more efficient management of these demands. This cycle of effective management then leads to an accumulation of additional resources. Conversely, when resources are scarce, the perception of demands becomes more burdensome, leading to less effective management and a heightened risk of resource depletion, leading to mental health difficulties.

Several predictors of school burnout both environmental (related to school or family environment) and personal have been investigated. At the school level, elevated school demands (i.e., authority conflict, emotional and mental demands), learning pressure, poor interpersonal relationships, and a negative teaching environment were positively related to burnout symptoms, while school resources (i.e., teacher support, control, autonomy) showed a negative association to burnout symptoms [20,21]. Concerning family factors, parenting style, family economic status, and parental support and acceptance have been found to influence burnout symptoms [20,22,23]. Further, elevated levels of life stress positively predict academic burnout in student samples [24].

Personal factors, which are the focus of the current study, refer to individual characteristics that positively or negatively predict students’ resilience in the academic context. Coping and coping flexibility has been found to negatively predict burnout symptoms [25,26,27]. Moreover, personality traits, temperament, and self-esteem have been identified to predict burnout symptoms [20,28,29].

Human cognitive functions such as learning, perception, attention, memory, reasoning, and problem solving are all significantly influenced by emotions [30,31]. An important construct that was extensively explored in relation to various mental health outcomes (see [32] for depression, [33] for psychotic symptoms), and which has recently received attention in relation to academic outcomes, including burnout [34,35], is emotion regulation (ER). According to Thompson [36], ER refers to “a series of intrinsic and extrinsic processes that are responsible for monitoring, evaluating and modifying emotional responses, especially in their temporal elements and intensity, in order to achieve personal goals”. ER strategies can be divided into adaptive or maladaptive, taking into account their affective, behavioral, and cognitive consequences [37]. The ER strategies can also be distinguished, according to the underlying activities, into cognitive and behavioral strategies [38].

In order to further understand the precise ER strategies that students use and how these, in turn, affect their academic achievement, there are recent studies that have explored this relationship [34,39,40]. Muchacka-Cymerman and Tomaszek [41] show that emotional coping strategies, both dispositional and situational, are negatively associated with overall burnout symptoms, while situational emotional coping can negatively influence symptoms such as inadequacy and a loss of interest in relation with school. Expressive suppression, which refers to attempts to reduce or inhibit ongoing emotion expressive behavior [42] has been shown to be positively associated with overall and specific burnout symptoms [35,43]. Further, emotional self-awareness has been linked to lower burnout rates in the workplace context [44].

Among the factors that can influence the development and usage of the ER strategies, attachment theory has been used to explore these variations, and attachment has received support as contributing factor [45,46,47]. According to Bowlby [48], human beings have an innate attachment behavioral system that motivates them to seek proximity to attachment figures. Interactions with significant others in times of need (e.g., stressful situations) that are met with care and availability stimulate the development of favorable internal working models that encompass positive beliefs about the availability of others and self-worth [49]. Internal working models further influence ER by guiding cognitive, affective, and behavioral responses in challenging situations. Thus, securely attached youth develop functional ER strategies on the basis of positive beliefs regarding their primary attachment figure’s availability and capacity to reduce their distress, as well as previous positive experiences regulating emotion with the assistance of caregivers [50,51,52]. 

Previous literature has shown that a secure attachment is associated with a confident, eager, attentive, and resourceful exploration of the environment, especially when the child is facing disappointment [53]. In emotionally provoking situations, the attachment system plays an important role in children’s support seeking. However, besides being an emergency response system, it also supports children’s exploration and self-regulatory capacities in everyday situations [54]). 

In middle childhood, attachment security is associated with more adaptive ER, manifested through less avoidance of conversing about negative emotions with mothers [55] and less difficulty identifying emotions [51]. In youth samples, the association between attachment security and adaptive ER is supported, with securely attached youth showing increased positivity, coherence of content and affect, lower emotion dysregulation [56], less dysfunctional anger [57], and the use of adaptive ER strategies [58]. Studies involving elementary school samples indicate that a stronger parent–child attachment negatively predicts learning burnout [59]. Moreover, studies conducted with university students demonstrate that an anxious attachment style positively predicts burnout symptoms [60,61]. 

The research of student burnout has been subjected to conflicting findings regarding male and female variations in burnout levels and symptoms expression. Unfounded hypotheses are commonly produced by a lack of understanding concerning gender differences and may incorrectly guide policy and clinical decisions. From a general point of view, taking into consideration the workplace burnout rates, there are slight differences in terms of burnout manifestation, with women showing slightly higher rates of emotional exhaustion, while men present higher rates of depersonalization [62]. Several studies show that in academic settings, girls experience higher levels of all three student burnout components [11,34]. On the contrary, other research shows that girls, on average, scored higher on the exhaustion subscale of school burnout than boys and there are no cynicism-related group mean differences [63].

The main aim of the current study is to examine whether emotion regulation strategies mediate the relationship between children’s attachment security and student burnout symptoms. A secondary objective of the study is to explore potential overall or symptom-specific differences in burnout levels between boys and girls.

## 2. Methods

### 2.1. Setting and Participants

Participants were enrolled through a larger research project on the validation of a game-based assessment system of emotion regulation skills in children and adolescents, carried out within the lab of the authors. The sample was comprised of 602 children (55% female) aged 8–16 (M = 10.45; SD = 2.14) from 18 schools. The students completed questionnaires assessing their level of attachment security, ER strategies, and levels of school burnout symptoms. In the current sample, 90.4% of students were enrolled in middle school (grades 2–8) and 15.5% were enrolled in private education programs.

### 2.2. Procedure

In September 2021, invitations for collaboration were sent via email to management bodies of schools from the county and to the County Center of Resources and Educational Assistance. Letters of informed consent were filled out by parents, either in physical format or online, through the REThink Emotions platform and app. All data were collected using the mobile app, in class, with the guidance of the project team.

### 2.3. Measures

#### 2.3.1. Student Burnout

The School Burnout Inventory (SBI [11]) consists of 9 items measuring three aspects of school burnout, namely exhaustion at school, cynicism toward the meaning of school, and the sense of inadequacy at school. All the items are rated on a 6-point Likert-type scale ranging from 1—completely disagree to 6—completely agree. 

The measure presents adequate psychometric properties, with a Cronbach’s alpha reliability for the overall school burnout scale ranging between 0.78 and 0.86, between 0.75 and 0.78 for exhaustion, between 0.83 and 0.87 for cynicism, and for inadequacy between 0.74 and 0.84 [11]. For the current sample, the Cronbach’s alpha for the emotional exhaustion subscale is 0.82, for cynicism it is 0.85, and for inadequacy it is 0.62.

#### 2.3.2. Emotion Regulation

The Emotion Regulation Index for Children and Adolescents (ERICA [64]) was used to assess ER. The first ERICA component (i.e., emotional control) involves the ability to modify your emotional responses and displays (e.g., I get angry when adults yell me what I can and cannot do—this item is reverse scored). The second ERICA factor (i.e., emotional awareness) contains items that represent emotional self-awareness (e.g., I am a happy person) and situational response (When adults are friendly with me, I am friendly with them). 

The measure includes 16 items rated on a 4-point scale (1 = never to 4 = almost always) and present adequate psychometric properties. For the current sample, the Cronbach’s alpha for the emotional control subscale is 0.79 and for emotional awareness it is 0.69. 

#### 2.3.3. Attachment Security

The Security Scale [65] is a self-report questionnaire developed to assess children and adolescent’s perception of security in parent–child relationships. Participants need to rate whether two statements for each of the items (i.e., “Some kids find it easy to trust their mom BUT Other kids are not sure if they can trust their mom.”) are ‘sort of true for me’ or ‘really true for me’. The scale offers a continuous measure of attachment security, with items addressing whether the child perceives the attachment figure as responsive and available.

The 15 item version of the measure exhibits adequate internal consistency across a number of samples, with the Cronbach’s alpha ranging from 0.64 to 0.93 when the targeted attachment figure is represented by the mother, and 0.81 to 0.88 when represented by the father [65,66,67,68,69,70]. For the current sample, the Cronbach’s alpha is one of 0.85.

### 2.4. Statistical Analyses

The study adopted a cross-sectional design. SPSS version 22.0 (International Business Machines Corporation–IBM, Armonk, NY, USA) was used to explore the sample’s characteristics. Pearson’s r correlation analyses were conducted to explore the associations between all the variables of interest (attachment security, emotion regulation strategies, and student burnout). In order to assess the reliability of the measurement tools, we employed Cronbach’s alpha. Analyses aimed at investigating the gender differences were conducted through independent t tests. Multiple regressions were used to explore the relationship between the independent variable, mediating variables, and dependent variables. Jamovi version 2.3.18 (The jamovi project, Sydney, Australia) was employed to perform path analysis modeling in order to test the mediation model. We used bootstrapping with 5000 samples to assess the significance of the standardized direct, indirect, and total effects of the variables influencing student burnout. 

## 3. Results

### 3.1. Associations between Attachment, Emotion Regulation, and Student Burnout

Descriptive statistics for the target variables can be consulted in Table 1. As presented in Table 2, negative significant correlations were identified between attachment security and exhaustion, r(599) = −0.50, *p* < 0.001, cynicism, r(599) = −0.51, *p* < 0.001, and inadequacy, r(599) = −0.47, *p* < 0.001 subscales of SBI, as well as between attachment and the overall burnout score, r(599) = −0.54, *p* < 0.001. Negative significant associations were also identified between the emotional control subscale of ERICA and exhaustion symptoms r(599) = −0.42, *p* < 0.001, cynicism symptoms, r(599) = −0.49, *p* < 0.001, and inadequacy symptoms, r(599) = −0.48, *p* < 0.001. The emotional awareness subscale was negatively associated with emotional exhaustion, r(599) = −0.57, *p* < 0.001, cynicism, r(599) = −0.56, *p* < 0.001, and inadequacy, r(599) = −0.55, *p* < 0.001. Positive significant correlations were identified between attachment security and emotional control, r(599) = 0.37, *p* < 0.001, as well as emotional awareness r(598) = 0.55, *p* < 0.001. 

### 3.2. Gender Differences

The results indicate that there are no significant gender differences between males and females concerning overall student burnout symptoms, as well as for specific subscales (see Table 3).

### 3.3. Model Estimation

(1) Direct associations between attachment, emotion regulation strategies, and overall burnout symptoms

The examination of the parameter estimates shows that all prediction pathways are significant. More specifically, attachment security significantly predicts functional ER strategies in children and adolescents. In turn, the use of functional ER strategies negatively predicts the onset of student burnout symptoms. For a more detailed assessment, please refer to Table 4.

(2) Indirect Effects

**Figure 1:**
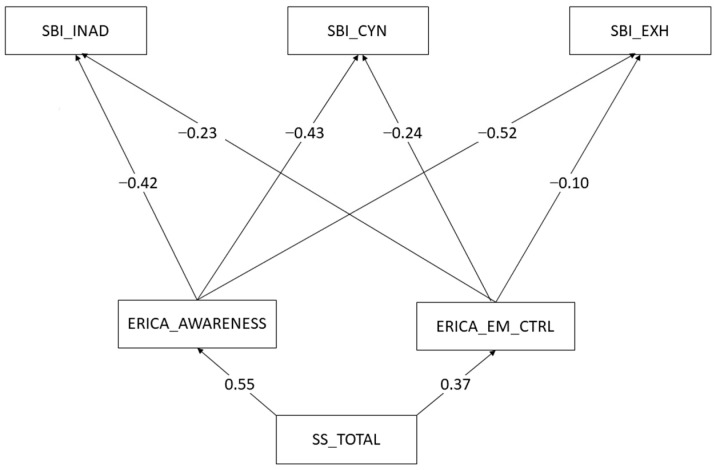
Path diagram. Note. The upper level of the figure presents the student burnout symptoms, followed by the emotion regulation strategies in the middle level and attachment security on the bottom level.

The hypothesis of the study proposes that the relationship between children’s attachment security and student burnout symptoms is mediated by ER strategies. The indirect effects computed based on the path analysis model (see Figure 1) reveal the presence of the expected effect for both emotional awareness and emotional control in the context of all burnout symptoms.

The relationship between attachment and emotional exhaustion is mediated by both emotional control and emotional awareness. This is also the case for cynicism, where the relationship is mediated by emotional control and emotional awareness. Finally, the relationship between attachment security and inadequacy in relation to schoolwork is also mediated by both emotional control and emotional awareness (see Table 5). 

## 4. Discussion

Given that student burnout is a widespread issue that can negatively influence academic outcomes and students’ wellbeing, the present research aimed to investigate its mechanisms, namely the relationship between attachment, burnout, and emotional regulation in a sample of children and adolescents. A secondary objective of the study was to explore the gender differences in burnout symptoms, following the line of research presented by previous studies that have explored the topic [34].

Our results have shown that both emotional awareness and emotional control negatively predict exhaustion, cynicism, and inadequacy symptoms, as well as overall student burnout scores in youth samples. In previous studies, ER strategies that have gained recognition as being adaptive have been, in general, negatively related to student burnout symptoms, whereas maladaptive ones have been positively related to these symptoms [70,71]. When it comes to the ER strategies investigated in the current paper, there are no studies relating either emotion awareness or emotional control to burnout symptoms in children and adolescents. Insight from studies conducted on adult samples suggests that higher emotion awareness is found in samples with low emotional exhaustion related to work activities [72] and the link between emotional self-awareness and burnout follows a negative trend [44]. Emotional control has also been negatively related to burnout symptoms in nurse samples [73]. 

The current findings, showing that emotional control and emotion awareness strategies for ER are significantly predicted by attachment security, further build on a long line of research that closely links attachment to ER in both children and adults [50,74]. Waters et al. [55] suggested that children with secure attachments are more willing to engage in talks about emotions and have a greater understanding of negative emotions. These findings are consistent with the current results, showing that children who are more securely attached have greater emotional awareness skills. Our findings on the relationship between attachment security and emotional control are also consistent with a comprehensive meta-analysis that showed that children and adolescents who are more securely attached experience less negative effects when provoked and are better at regulating emotions [52]. 

Lower attachment security has been previously linked to student burnout symptoms in college samples [61] as well as work-related burnout in adult samples [75,76] (Leiter et al., 2015; West, 2015). On the contrary, a higher level of attachment security has been negatively related to emotional exhaustion and depersonalization burnout symptoms, while being positively related to students’ personal accomplishment [77]. Our paper extends these findings to youth samples, showing that a secure attachment negatively predicts exhaustion, cynicism, and inadequacy symptoms in children and adolescents.

The results of the path analysis show that both emotional awareness and emotional control mediate the relationship between attachment security and student burnout symptoms, supporting our main hypothesis. These findings provide insight on a potential mechanism in the relationship between attachment security and burnout, and are in line with the extensive research that emphasizes the role of children’s secure attachment for their emotional health [56,78]. Furthermore, our findings indicate that a more secure attachment can also positively reflect on children’s well-being in an educational setting. Categorically, more complex studies need to be carried out in order to further explore the mechanisms of this relationship and acknowledge the role of various environmental variables that might be contributing to this observed outcome. Factors such as classroom dynamics, teacher-student interactions, peer relationships, and broader educational environments could play pivotal roles in shaping how secure attachment translates into enhanced well-being among students.

Although previous studies and meta-analyses indicated gender differences concerning burnout symptomatology, we were unable to replicate these findings. The current study was unable to identify gender differences in terms of overall or symptom-specific burnout levels. Cultural studies show that in the school context, collectivistic cultures prioritize interpersonal relationships, interdependence, and intragroup ambitions [79]. A possible explanation for the diverging results might arise from the cultural context of the study, as the sample comes from a previously collectivistic society. Future studies should investigate cultural and social influences that could explain the difference in symptom expression identified within student burnout research.

One important limitation of this study is that the children were predominantly enrolled in public schooling. Studies have shown higher levels of academic stress being present in public education institutions [80]. Given the well-established relationship between academic stress and burnout [81], future studies should investigate how school characteristics could affect burnout rates.

Academic stress can fluctuate as a function of age, with older children experiencing higher levels of stress in face of exams and growing demands [82]. Being that the current sample mainly consists of middle school students, age differences concerning burnout rates were unable to be established.

An additional limitation is important to note. Self-report measures were completed by children for attachment, ER strategies, and burnout levels. All the measures are susceptible to desirability, with children being evaluated in classrooms. Taking into account the measure of ER, the limited number of ER strategies included may limit the information concerning possible strategies employed by children in the context of academic stress.

Future studies should consider sampling methods that could assure a more balanced sample in terms of age and school characteristics, which would allow more differences in terms of student burnout levels to be established. In terms of assessment, given that burnout symptoms can fluctuate as a function of stress levels, it is recommended that Ecologic Momentary Assessment tools be used in order to have a better understanding of the development of student burnout symptoms.

From a practical standpoint, the current study brings to attention the importance of warm and reliable parental involvement as a foundation for the development of secure attachment in children which would further encourage the use of adaptive ER strategies and a better awareness and understanding of emotional states. These prerequisites have the potential to act as a buffer in the development of burnout symptoms in highly stressful academic settings.

## 5. Conclusions

Establishing the role of emotional awareness and emotional control as a precursor to school burnout has implications for school counselors, psychologists, and educators alike. Strategies guided by the present results would aim at increasing the use of emotional awareness and emotional control in the context of academic challenges posed by high requirements and stress levels. 

Students who appear to be experiencing stress connected to school requirements might benefit from one-on-one or group counseling sessions with school psychologists and counselors who need to educate them about the use of adaptive ER strategies. Several randomized clinical trials present various approaches to training emotion regulation through counseling activities. The content of the sessions can include meditation, breathing exercises, imagination exercises, raising awareness to one’s thoughts (i.e., encouraging youth to track their thoughts by keeping a diary), and providing psychoeducation about characteristics and emotional expressions in order to improve emotional awareness and control. Further, testing the validity of thoughts through finding evidence that supports change, Socratic questioning, the formulation of alternative thoughts, setting emotion-regulation goals, and acceptance all aim to improve control over impulsive behavior and provide a lasting change in the emotion regulation abilities of youth [83,84,85]. 

## Figures and Tables

**Table 1:** Descriptive statistics.

	N	Minimum	Maximum	Mean	Std. Deviation
SBI_EXH	602	4	24	9.56	4.962
SBI_CYN	602	3	18	6.58	3.983
SBI_INAD	602	2	12	5.03	2.694
ERICA_EM_CTRL	602	7	35	24.02	5.636
ERICA_AWARENESS	602	4	20	15.32	3.333
SS_TOTAL	602	18	60	49.78	8.554

**Table 2:** Pearson correlations (two-tailed) between attachment security, emotion regulation strategies, and burnout.

	1	2	3	4	5	6	7
ERICA_EM_CTRL	*—*						
ERICA_AWARENESS	0.628 ***	—					
SS_TOTAL	0.372 ***	0.549 ***	—				
SBI_EXH	−0.421 ***	−0.573 ***	−0.497 ***	—			
SBI_CYN	−0.488 ***	−0.558 ***	−0.506 ***	0.755 ***	—		
SBI_INAD	−0.479 ***	−0.545 ***	−0.471 ***	0.751 ***	0.714 ***	—	
SBI_TOTAL	−0.501 ***	−0.614 ***	−0.541 ***	0.940 ***	0.908 ***	0.872 ***	*—*

Note. ERICA_EM_CTRL = Emotional Control; ERICA_AWARENESS = Emotional Awareness; SS_TOTAL = Attachment Total; SBI_EXH = Exhaustion; SBI_CYN = Cynicism; SBI_INAD = Inadequacy; SBI_TOTAL = Total Burnout. *** *p* < 0.001.

**Table 3:** Gender differences in student burnout levels.

	*T*	*df*	*p*
SBI_EXH	−0.170	599	0.865
SBI_CYN	1.298	599	0.195
SBI INAD	0.462	599	0.644
SBI_TOTAL	0.524	599	0.600

Note. SBI_EXH = Exhaustion; SBI_CYN = Cynicism; SBI_INAD = Inadequacy; SBI_TOTAL = Total Burnout.

**Table 4:** Direct associations between attachment security, emotion regulation, and student burnout.

Parameter Estimates								
				95% Confidence Intervals				
Dep	Pred	b	SE	Lower	Upper	β	z	*p*
SBI_EXH	ERICA_AWARENESS	−0.7585	0.0662	−0.887	−0.629	−0.522	−11.45	<0.001
SBI_EXH	ERICA_EM_CTRL	−0.0884	0.0398	−0.166	−0.010	−0.102	−2.22	0.026
SBI_CYN	ERICA_EM_CTRL	−0.1606	0.0323	−0.225	−0.098	−0.237	−4.97	<0.001
SBI_CYN	ERICA_AWARENESS	−0.4957	0.0587	−0.608	−0.376	−0.434	−8.44	<0.001
SBI_INAD	ERICA_EM_CTRL	−0.1075	0.0222	−0.149	−0.064	−0.234	−4.84	<0.001
SBI_INAD	ERICA_AWARENESS	−0.3266	0.0374	−0.401	−0.252	−0.421	−8.73	<0.001
ERICA_EM_CTRL	SS_TOTAL	0.2464	0.0243	0.198	0.294	0.370	10.14	<0.001
ERICA_AWARENESS	SS_TOTAL	0.2159	0.0141	0.188	0.243	0.547	15.28	<0.001

Note. ERICA_EM_CTRL = Emotional Control; ERICA_AWARENESS = Emotional Awareness; SS_TOTAL = Attachment Total; SBI_EXH = Exhaustion; SBI_CYN = Cynicism; SBI_INAD = Inadequacy.

**Table 5:** Indirect effects.

			95% Confidence Intervals				
	b	SE	Lower	Upper	β	z	*p*
SS_TOTAL ⇒ ERICA_EM_CTRL ⇒ SBI_CYN	−0.039	0.010	−0.059	−0.022	−0.087	−4.109	<0.001
SS_TOTAL ⇒ ERICA_EM_CTRL ⇒ SBI_EXH	−0.022	0.011	−0.043	−0.002	−0.038	−2.058	0.040
SS_TOTAL ⇒ ERICA_EM_CTRL ⇒ SBI_INAD	−0.026	0.007	−0.040	−0.014	−0.086	−3.949	<0.001
SS_TOTAL ⇒ ERICA_AWARENESS ⇒ SBI_CYN	−0.107	0.015	−0.137	−0.077	−0.237	−7.038	<0.001
SS_TOTAL ⇒ ERICA_AWARENESS ⇒ SBI_EXH	−0.164	0.019	−0.203	−0.128	−0.285	−8.464	<0.001
SS_TOTAL ⇒ ERICA_AWARENESS ⇒ SBI_INAD	−0.071	0.010	−0.090	−0.052	−0.230	−7.301	<0.001

Note. ERICA_EM_CTRL = Emotional Control; ERICA_AWARENESS = Emotional Awareness; SS_TOTAL = Attachment Total; SBI_EXH = Exhaustion; SBI_CYN = Cynicism; SBI_INAD = Inadequacy.

## Data Availability

The data that support the findings of this study are available from the corresponding author, upon reasonable request.
